# Vascular Endothelial Growth Factor Ligands and Receptors in Breast Cancer

**DOI:** 10.3390/jcm12062412

**Published:** 2023-03-21

**Authors:** Klaudia Katarzyna Brogowska, Monika Zajkowska, Barbara Mroczko

**Affiliations:** 1 Public Health Care Hospital in Knyszyn, 19-120 Knyszyn, Poland; 2 Department of Neurodegeneration Diagnostics, Medical University of Bialystok, 15-269 Bialystok, Poland; 3 Department of Biochemical Diagnostics, Medical University of Bialystok, 15-269 Bialystok, Poland

**Keywords:** breast cancer, VEGF, diagnosis, tumor markers, angiogenesis

## Abstract

Breast cancer (BC) is the most common malignancy responsible for the largest number of deaths in women worldwide. The risk of developing BC is predisposed by many factors such as age, presence of genetic mutations or body weight. The diagnosis is mostly made relatively late, which is why patients are exposed to radical surgical treatments, long-term chemotherapy and lower survival rates. There are no sufficiently sensitive and specific screening tests; therefore, researchers are still looking for new diagnostic biomarkers that would indicate the appearance of neoplastic changes in the initial stage of neoplasm. The VEGF family of proteins (VEGF-A, VEGF-B, VEGF-C, VEGF-D, EG-VEGF, PlGF) and their receptors are significant factors in the pathogenesis of BC. They play a significant role in the process of angiogenesis and lymphangiogenesis in both physiological and pathological conditions. The usefulness of these proteins as potential diagnostic biomarkers has been initially proven. Moreover, the blockage of VEGF-related pathways seems to be a valid therapeutic target. Recent studies have tried to describe novel strategies, including targeting pericytes, use of miRNAs and extracellular tumor-associated vesicles, immunotherapeutic drugs and nanotechnology. This indicates their possible contribution to the formation of breast cancer and their usefulness as potential biomarkers and therapeutic targets.

## 1. Breast Cancer

### 1.1. Epidemiology

**Figure 1:**
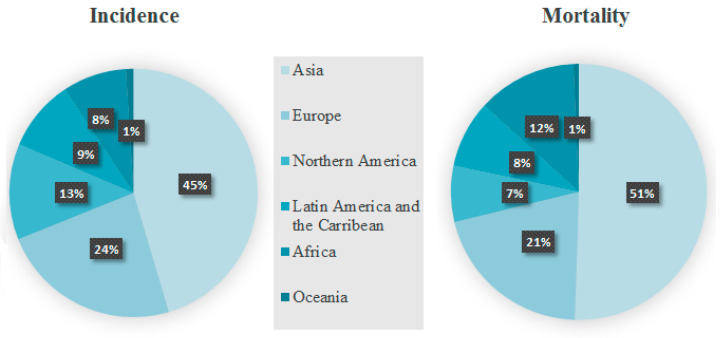
Breast cancer incidence and mortality in 2020 for women [1].

Based on the studies published so far, the prevalence of breast cancer is determined significantly by ethnicity and is higher in developed countries. However, breast cancer mortality is higher in less-developed regions. According to the Global Cancer Observatory, there were 2,261,419 new cases of breast cancer worldwide, which accounts for 11.7% of the total number of cancer cases in the population in 2020 [1,2]. The most of breast cancer cases were recorded in East Asia (551,636), North America (281,591), South-Central Asia (254,881), Western Europe (169,016), Southeast Asia (158,939), Central and Eastern Europe (158,708), South America (156,472) and Southern Europe (83,177). Interestingly, the majority of reported cases were reported in Asia (45.4%), followed by Europe (23.5%) and North America (12.5%). What is more, 684,996 patients died from breast cancer, which corresponds to 6.9% of all cancer deaths in the course of the same year. Furthermore, the highest number of deaths was recorded in East Asia (141,421), South Central Asia (124,975) and South East Asia (58,670) (Figure 1) [1].

**Figure 2:**
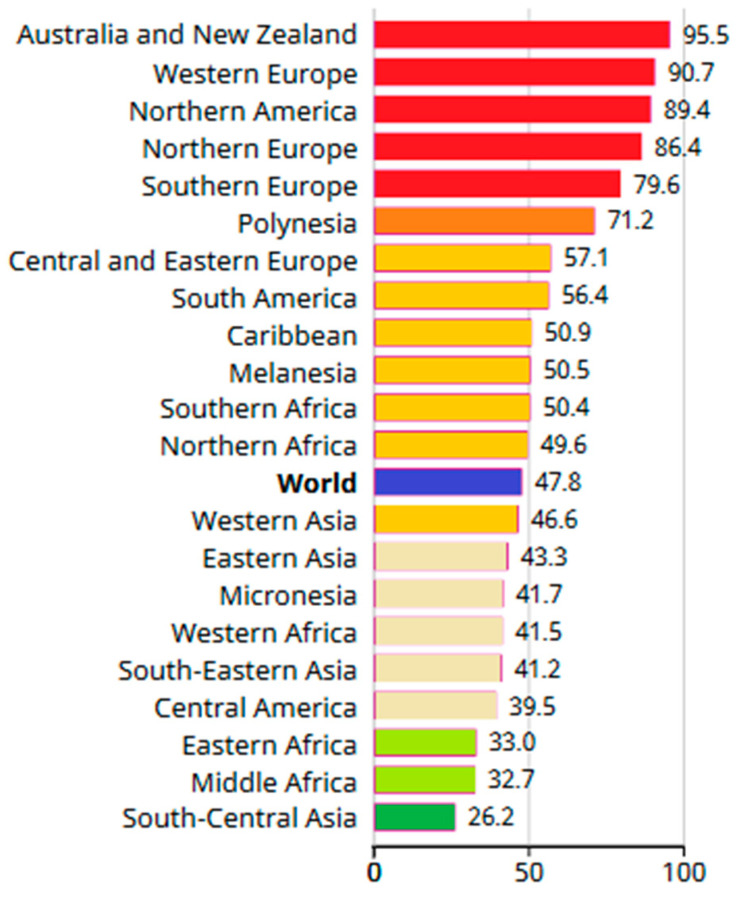
Breast cancer age standardized rate (ASR per 100,000) in 2020 for women [1].

The risk of developing breast cancer increases with age; therefore, the age standardized rate (ASR—weighted average of the age-specific mortality rates per 100,000 persons) is usually used in statistics. In 2020, the ASR was 95.5 for Australia and New Zealand, while in North America, Western, Northern and Southern Europe, it was above 79.5. More detailed information on ASR is provided in Figure 2. The highest standardized mortality rates (SMR—ratio of observed number of deaths in the cohort to the number of deaths that would be expected) were recorded in Polynesia (22.3), Melanesia (27.5), the Caribbean (18.9), North (18.8) and West Africa (22.3). It was lowest in East Asia (9.8) and Central America (10.4). Such low survival rates in these countries mainly result from the delay in the diagnosis and poor access to treatment for patients with breast cancer [1].

### 1.2. Pathogenesis

Breast cancer is characterized by the uncontrolled growth of cells, their invasion and, eventually, their spread from their origin to distant locations. Neoplastic changes may affect different types of tissues, which means that each type of breast cancer has its own, specific characteristics. The changes that lead to the development of cancer are commonly known as carcinogenesis [3]. Cancer cells are characterized by high genomic instability, metabolic reprogramming and the ability to avoid destruction by the host’s immune system [3].

It has been shown that inflammation is related to the initiation, growth and metastasis of cancer—it intensifies cell proliferation, which contributes to mutations, as well as intensifies the production of compounds responsible for DNA damage [4]. Macrophages, the presence of which can be observed in the tissues of the mammary gland, participate in its physiological development and remodeling. While macrophages are being activated, they become one of the most important cells of inflammatory processes due to their intensive production of cytokines, mostly chemokines and growth factors, including VEGF (Vascular Endothelial Growth Factor), which stimulates the formation of new blood vessels. Macrophages found in increased numbers in neoplastic tissue have been termed tumor-associated macrophages (TAMs). It has been observed that TAMs, instead of having a protective function, facilitate the spread of cancer cells [5,6]. Primary tumor macrophages, stimulated by IL-4 secreted by CD4+ T lymphocytes and neoplastic cells, escalate the progression of disease and suppress cytotoxic T-cell response. In case of breast cancer, this results in a dynamic progression of proliferative changes through an increased supply of nutrients and oxygen to pathological cells and the possibility of their transfer to peripheral vessels [5,7].

Hormones and their receptors also play an important role in the pathogenesis of breast cancer. In the case of genetic predisposition, such as a mutation within the BRCA1 and BRCA2 genes, the relevance of hormones in breast cancer development increases significantly [8,9]. Estrogens may lead to the development of de novo breast cancer lesions. By binding to receptors, estradiol increases cell proliferation, which is associated with an increased risk of errors in replication and the formation of mutations that, due to the rate of cell division, may not be repaired and will be passed on. The clinical importance of ER-α in breast cancer has been confirmed, while the role of ER-β is not fully understood. The mature mammary gland has a higher level of estradiol and progesterone compared to the immature gland, which correlates with accelerated cell proliferation [8,9]. There are a large amount of substances capable of mimicking estrogen in the environment, such as polychlorinated biphenyls and organochlorine pesticides, which can penetrate the breast tissue and have estrogen-like effects [8,9]. Expression of the PR (progesterone) gene is controlled by estrogen; therefore, the detected PR expression is believed to indicate an intact estrogen–ER pathway. For this reason, PR expression is used as a biomarker to evaluate ER-α function and has a prognostic value: breast cancer with ER(+) PR(+) phenotype shows higher survival rate than in ER(+) PR(−) [8,9,10]. Similarly, overexpression of HER2 (Human epidermal growth factor receptor-2) in breast cancer cells is closely related to the proliferation of neoplastic cells and the progression of the carcinogenesis process, and its pro-carcinogenic effect results from the intensification of inflammation and increased expansion of tumor stem cells (CSCs) [11,12]. EGFR (Epidermal growth factor receptor) overexpression has been detected in almost 1/3 of patients with highly aggressive inflammatory breast cancer (IBC) and in more than half of patients with triple-negative breast cancer (TNBC), which indicates a poor prognosis [11].

### 1.3. Risk Factors

Reproductive, environmental and lifestyle factors influence the development of breast cancer. All these causes can be divided into non-modifiable factors, which include gender, age, race, family history and genetic mutations, and modifiable factors, which include eating habits, weight or amount of adipose tissue. Hereditary genetic mutations account for approximately 10% of all breast cancers [13,14].

Breast cancer mainly affects women, about 1% of cases occur in men. In men, the main cause is disorder of the endocrine function of the testicles, as well as genetic mutations predisposing to the disease, including a mutation in the BRCA2 gene (breast cancer 2 gene). Other known risk factors in males include Klinefelter syndrome, obesity, exposure to ionizing radiation, positive family history and the presence of benign breast diseases [13,14].

The risk of developing breast cancer significantly increases with age and reaches its peak in menopause, then gradually decreases or remains at a similar level due to the accumulation of mutations [2,13]. A significant correlation was observed between the age at which the neoplastic disease was diagnosed and the expression of estrogen receptor in the examined cancer tissue. Neoplasms with overexpression of the estrogen receptor (ER(+)) are characterized by an increased incidence with age, while ER(−) tumors are more common in patients under 50 years of age [2,13].

Reproductive factors such as the early onset of menstruation, late entry into menopause, and late first pregnancy may also increase the risk of breast cancer. Every year delaying the onset of menopause increases the risk of menopause by 3%, while each year of delaying the onset of *menarche* reduces the risk by 5% [11].

Another risk factor for developing breast cancer is a positive family history. Detection of breast cancer in a first-degree relative is associated with a three-fold higher risk of developing breast neoplasm [13].

Over the last decades, many studies have been carried out to identify specific mutations in genes whose are linked to an increased risk of breast cancer. The most important of them are BRCA1 and BRCA2, TP53 (tumor protein 53), PTEN (phosphatase and tensin homolog gene), STK11 (serine/threonine kinase 11 gene), NF1 (neurofibromatosis type 1) and CDH-1 (cadherin-1 gene) [15]. Moderate risk mutations in other genes affecting malignant transformation have also been highlighted: CHEK2 (effector kinase gene), PALB2 (BRCA2 protein stabilizer gene), RAD51C (RAD51 homolog C (*S. cerevisiae*)), NBN (previously known as NBS1), BRIP1 (protein gene that interacts with BRCA1), ATM and genes determining the occurrence of Lynch syndrome (MLH2, MSH1) [13,14,15]. According to the leading medical societies, such as the European Society of Medical Oncology (ESMO), The National Institute for Health and Care Excellence (NICE) and The National Comprehensive Cancer Network (NCCN), to confirm mutations in people at higher risk, it is necessary to perform genetic testing to detect a specific mutation [13,14].

One of the fundamental, modifiable risk factors for breast cancer is obesity and poor eating habits. Consuming high-fat and highly processed foods promotes cancerous processes. Furthermore, high levels of insulin and insulin-like growth factors (IGFs) stimulate the proliferation of cancer cells. Chronic hyperinsulinemia reduces the levels of IGF binding proteins and SHBGs (sex hormone binding globulins), thereby increasing their concentration. Obesity increases the conversion of androgen precursors to estrogens through peripheral aromatization in adipose tissue [2,13,14].

### 1.4. Clinical Symptoms

**Figure 3:**
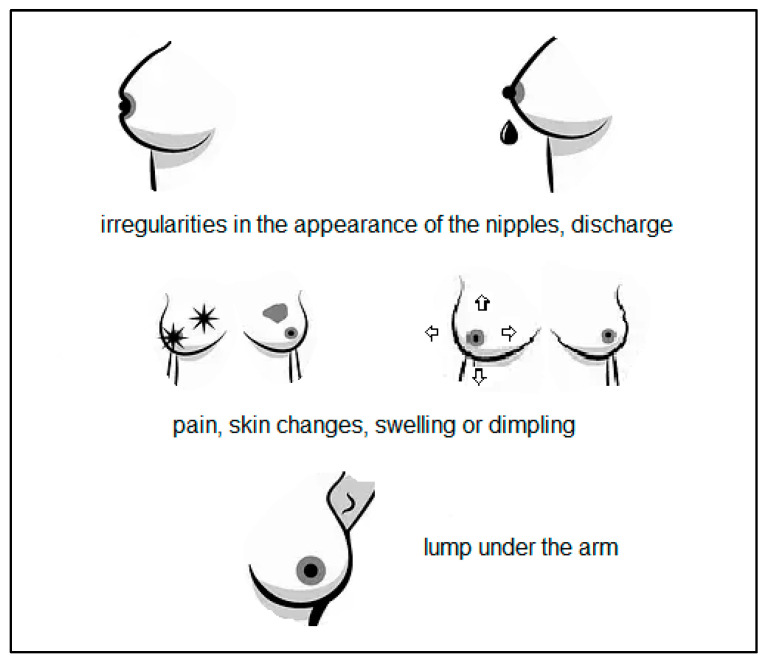
Most commonly observed breast cancer clinical symptoms.

The most common symptoms that occur in about 80% of women diagnosed with breast cancer are shown in Figure 3 [16,17]. Some women also report to a specialist for further diagnosis due to disturbing ulceration or rash, infection or inflammation of the mammary gland and changes in the shape or asymmetry of the breasts. It is common to enlarge both the axillary and the cervical lymph nodes [16,18]. There are cases in which patients do not report for 90 days after the onset of symptoms, which contributes to a worse prognosis [18]. Patients may also have non-specific symptoms, including back pain, skeletal and muscular pain, shortness of breath, chest pain, abdominal pain, fatigue and weakness, weight loss, cough, upper limb swelling and bruises appearing on the breasts without any specific cause [16].

### 1.5. Classification

Breast cancers are a heterogeneous group with a large variety of morphological features, which translates into the choice of appropriate diagnostic and therapeutic procedures and differences in response to treatment. Thanks to the introduction of the appropriate classification, it is possible to make a more accurate diagnosis and establish personalized therapy. The basic steps in the assessment of breast cancer are based on the diagnosis of the type of cancer, its classification and staging based on the guidelines of the World Health Organization (WHO). In order to determine the stage of breast cancer, both the size of the tumor and the condition of the lymph nodes should be assessed, and the possible presence of distant metastases should be checked, and the presence of ER, PR and HER2 receptors should be taken into account [19]. Currently, the latest classification based on molecular biology and expression of the studied genes is used [20]. Current molecular classification divides breast cancer into groups due to the expression of individual genes for luminal A and B, HER2/neu and basal-like. Lately, the HER2 subgroup has been additionally divided into three clinically different groups. All of the previously mentioned subgroups differ in terms of patient prognosis, treatment response, clinical and biological properties [20].

Histological classification is complex and includes many types of cancer, but the most important is their division into invasive and pre-invasive. In the case of ductal carcinoma in situ limited to the mammary ducts, metastasis should not occur. Lobular carcinoma in situ affects the lobular-alveolar segment and may increase the risk of invasive cancer. The most common histological diagnoses are: infiltrating ductal carcinoma (75%), infiltrating lobular carcinoma (10–15%), tubular carcinoma (5%), medullary carcinoma (5%) and mucinous carcinoma (5%) [19,21]. One of the most popular classifications, which was previously used for many types of cancers, is the TNM classification [21].

### 1.6. Diagnostics and Prevention

For the early detection of breast cancer, it is recommended to screen and report the first disturbing symptoms to a doctor, which will allow for quick diagnosis and treatment. Any disturbing changes detected during breast self-examination should be reported to a specialist as soon as possible to perform a palpation examination by a specialist and to perform additional tests [22,23]. In addition to palpation, other screening methods such as X-ray mammography, also known as the gold standard of breast imaging, which reduces overall mortality by up to 20%, can be performed [23]. During the procedure, digital cameras are used, less often analog or digitized. Digital mammography can be extended by tomosynthesis (DBT) or performed with contrast enhancement (CESM)—both possibilities allows for better assessment of doubtful and ambiguous lesions in a routine examination and the assessment of the multifocality of a possible neoplasm [24]. For carriers of BRCA1, BRCA2 and other mutations, additional supplementary examination in the form of MRI (magnetic resonance imaging) is used, which allows the detection of small neoplastic changes that would not be noticeable in a mammography. MRI screening can also be used in patients after breast augmentation surgery, in whom routine screening is not possible and the results of other tests have been inconclusive [15,23]. Some sources claim that the third examination that can be used in screening is breast ultrasound, but it is usually performed when the mammography results are inconclusive and MRI cannot be performed. Ultrasound is also used in young women whose breasts are dominated by glandular tissue [15,23]. The frequency of follow-up in asymptomatic women with no risk factors for breast cancer is presented in Table 1.

For early diagnosis, a clinical interview is carried out, in which information about the patient’s health and accompanying symptoms are collected, and a palpation examination is performed. Information on the menstrual cycle, menopause and postmenopausal hormonal treatment, pregnancies, contraception used, family history of cancer and comorbidities play an important role. After collecting the history and assessing the symptoms observed by the patient, the doctor performs a physical examination to capture and describe any abnormalities such as asymmetry, changes in the nipples and skin, palpable lumps or enlarged lymph nodes [15,22,25]. Specialized examinations, most often mammography in two projections in older women and ultrasound in younger women, are performed. Ultrasonography can be also used as a confirmation test to mammography, as it enables the differentiation between cysts and solid tumors and allows for an accurate assessment of the observed changes size. If an anomaly is detected, another control examination can be recommended after a specified time, for example the extraction of atypical tissue or histopathological examination with use of fine-needle aspiration (FNA) or core-needle (CNB) biopsy [22]. In patients with a genetic mutation, in justified cases, magnetic resonance imaging is also performed, which plays an important role in assessing the advancement and extent of changes, detecting possible neoplastic infiltration and selecting the appropriate treatment [22,23]. If an early stage of cancer is suspected—lung X-ray and routine blood laboratory tests are performed. In case of highly advanced cancer suspicion—CT of the chest, abdominal cavity and PET/CT with sodium fluoride can be implemented. After the diagnosis is made and the type of cancer is determined, the appropriate treatment is selected [23].

Currently, in breast cancer diagnosis mammography, ultrasound, in combination with biochemical markers such as CEA, CA 15-3 and CA 27.29 are used. Carcinoembryonic antigen (CEA) is considered as a most useful biomarker for colorectal cancer. It shows low diagnostic sensitivity and specificity, but its concentration increases in advanced stage of breast cancer, often when metastases are present; therefore, it cannot be used as a screening test. It can be used for breast cancer relapse monitoring due to its positive predictive value (>90%) [26,27]. CA 15.3 and CA 27.29 are two different epitopes of the same mucin (MUC1) gene. As a result of neoplastic transformation, MUC1 is overexpressed, and CA 15-3 and CA 27.29 are released from damaged epithelial cells to the bloodstream. Similarly to the CEA, the sensitivity and specificity of CA 15-3 in non-advanced breast cancer is low; thus, this protein cannot be used in screening. Increased CA 15-3 concentration is especially observed in patients with advanced disease and, therefore, it is used to detect recurrence and metastases [28,29]. It has been shown that simultaneous measurements of CEA and CA 15-3 might be important for the early detection of disease recurrence [30]. Combining CEA and CA 15-3 assessments with the evaluation of other factors such as HER2, patient age, TNM, expression of ER and PR may allow for a more accurate risk score of metastasis and mortality in young patients after tumor resection surgery [30,31,32]. Another promising protein is Ki-67, the expression of which allows for the assessment of the proliferation rate of neoplastic cells and may be used in the estimation of the remission period in the future. Its overexpression despite the early stage of neoplastic disease may suggest the need for chemotherapy implementation [26,27]. ER and HER2 receptors are important in the diagnosis of breast cancer. Estrogen receptor expression is detected in 70% of breast cancer cases—the α isoform is more often measured, but the β isoform is supposed to have a better prognosis for treatment and remission period. In patients with ER+ breast cancer, hormonal treatment is more effective. HER2 overexpression occurs in 10–35% of breast cancer patients and is unfavorable, as it is associated with an aggressive course, greater chance of metastases to the brain and worse prognosis [26]. Considering low sensitivity and specificity of the markers used so far, especially in early stages of breast cancer, researchers are looking for better biomarker that could be used in screening. There are many studies that assess the potential diagnostic importance of various molecules, including matrix metalloproteinases (e.g., MMP-9), nuclear proteins (e.g., PCNA), chemokines (e.g., CCL2 and CCL5), caveolins (e.g., CAV1 and CAV2), ATIP3 protein and others [33,34].

## 2. VEGF Family

### 2.1. Ligands

The family of vascular endothelial growth factors, which are the most potent pro-angiogenic factors, include secreted proteins: VEGF-A, VEGF-B, VEGF-C, VEGF-D and placental growth factor (PlGF). Recently, endocrine-derived vascular endothelial growth factor (EG-VEGF) has also been included in this group of parameters. In humans, the VEGF gene at locus 6p21.3 is part of a group of genes that encode a cluster of proteins called the cystine-knot growth factor superfamily, which also includes PDGF, NGF, and TGF-β. VEGF is a heterodimeric glycoprotein in which the cystine node has a characteristic disulfide bridge pattern [35,36,37].

Vascular endothelial growth factor A (VEGF-A) is the most prevailing factor inducing both angiogenesis and vasculogenesis. The gene encoding the VEGF-A factor contains eight exons and seven introns; exons 1–5 have an identical structure in each of the isoforms. It is assumed that exons 6 and 7 determine affinity for heparin, and exon 8 is associated with the proliferation of endothelial cells [35]. The VEGF-A isoforms include: VEGF121, VEGF145, VEGF148, VEGF162, VEGF165, VEGF165b, VEGF183, VEGF189 and VEGF206. All isoforms differ in length and biological properties. Each of the above-mentioned isoforms is important for the course of angiogenesis, but they differ in their role. The dominant isoform is VEGF165, which is lacking exon 6. It has a moderate affinity for heparin, thanks to which, it remains bounded to endothelial cells. The second most frequent isoform is VEGF121, lacking both exon 6 and 7. VEGF189 and VEGF206 are the longest isoforms with a strong affinity for heparin, which explains their weakest activity. VEGF-A binds to the VEGFR-1 receptor, and weaker to VEGFR-2. Additionally, VEGF165 has the ability to bind to the NP-1 and NP-2 receptors, and VEGF145 to NP-2. VEGF-A is secreted to the greatest extent by endothelial cells. In a state of hypoxia, this ability is also acquired, among others, by thrombocytes, macrophages, dendrocytes, astrocytes, osteoblasts, lymphocytes and tumor cells [35].

There are two isoforms of VEGF-B: VEGF-B167, which is the dominant form, and VEGF-B186. The first one has a molecular weight of 21 kDa, the ability to bind to the surface of cells or elements of the extracellular matrix, and an affinity for VEGFR-1 and NP-1 receptors [35]. The second isoform has a mass of 32 kDa and, unlike VEGF-B167, is occurring in free form. VEGF-B186 activates VEGFR-1 but requires prior proteolysis to bind to NP-1. Under physiological conditions, increased VEGF-B expression was observed in skeletal and heart muscle cells and in the pancreas [35].

VEGF-C promotes lymphangiogenesis by strongly activating VEGFR-3 receptors. At the same time, it shows lower affinity to VEGFR-2, the activation of which has a weak effect on angiogenesis [35,37]. More recent sources indicate that the mechanism of action of VEGF-C is probably associated with binding to NP-2, which influences the enhancement of VEGFR-3 activity [35,37]. VEGF-D has similar properties to VEGF-C, however, it binds to the VEGFR-3 and NP-2 receptors, affecting only the lymphangiogenesis process [35,37]. The newest member of the VEGF family, EG-VEGF, is called prokineticin (PK1) and has an affinity for the PROKR1 and PROKR2 prokineticin receptors. It was firstly detected in endocrine cells such as the testes, ovaries, adrenal glands and the placenta [35,37]. The last of the VEGF family members is PlGF. Its four isoforms differing in properties and structure are known—PlGF-1 (PlGF131), PlGF-2 (PlGF152), PlGF-3 (PlGF203) and PlGF-4 (PlGF224). Each of them is capable of binding to VEGFR-1. Additionally, PlGF-2 has the ability to bind to NP-1, NP-2 and heparin of the extracellular matrix [35].

#### 2.1.1. Ligands Physiological Role

VEGF-A is binding to VEGFR-1 and VEGFR-2 receptors on the endothelial surface, by which it regulates the proliferation and migration of endothelial cells, as well as vascular permeability and activation of inflammatory cells (macrophages and granulocytes) and tip cells. Those effects are based on the secretion of cytokines, chemokines and other growth factors influencing regenerative and angiogenesis processes. VEGF-A determines the maintenance of homeostasis of internal organs, including regenerative capacity of the liver by releasing HGF, HB-EGF, CTGF, IL-6 which enhance hepatocyte proliferation, and also enables lung regeneration by inducing the release of MMP-14, which also leads to an increase in cell proliferation [35,38]. Furthermore, VEGF-A is instrumental in the regulation of neural progenitor cell proliferation, as well as axonal survival, migration and specialization, as well as synaptic function [39]. VEGF-A is highly expressed by human podocytes in situ in developed, healthy adult kidney, although no angiogenesis is observed in healthy glomeruli. In human podocytes, VEGF-A plays an autocrine role in podocyte survival through interaction with nephrin. Retinal pigment epithelial (RPE) cells secrete growth factors, including VEGF-A, which plays an autocrine and paracrine role and is involved in maintenance of the choroidal endothelium and stabilization of the choroidal fenestrae. VEGF-A plays an essential role in regulation of fatty acid uptake. By mediating activation of VEGFR-2 it upregulates fat acids binding protein (FABP4) in the endothelium, which is involved in the trafficking and storage of fat acids [40].

VEGF-B has a significant impact on the proper shaping of the cardiovascular system and the heart muscle itself in the womb. The role of VEGF-B in angiogenesis is believed to be less important than VEGF-A, and its most important function under physiological conditions is to enable the survival of smooth muscle cells, neurons, pericytes, myocardial cells and vascular endothelial cells [35]. In most of the studies performed to date, VEGF-B does not appear to induce capillary angiogenesis, but does have an effect on myocardial capillary enlargement and coronary artery growth. Moreover, it shows cardioprotective activity by counteracting apoptosis of cardiomyocytes and a positive effect on the contractility of the heart muscle. In addition to the effect of VEGF-B on the angiogenesis process by activating VEGFR-1 receptors and its key importance in embryonic development, the latest research also indicates its protective effect on neurons and the heart muscle as well as its impact on metabolism [41]. Studies suggest that VEGF-B influences the expression of FATP on the endothelium and, thus, the transport of fatty acids by LCFA uptake [40]. VEGF-B was expressed in Müller cells at the highest levels compared with other members of the VEGF family. Recombinant VEGF-B restored Müller cell glutamine synthetase expression under hypoxic conditions and protected Müller cells [42].

High VEGF-C expression can be observed during embryonic development in the formation of lymphatic vessels, as well as in adults in the cells of the heart, thyroid, ovary, placenta and intestine [35,37]. Some studies have shown that VEGF-C plays its role in the maintenance of glomerular function. Moreover, it possibly promotes the survival of the uterine vascular endothelium along with VEGF-A and PlGF. Similarly to the rest of the family, VEGF contributes to the physiological development of the lungs, but its role is not fully understood [40].

VEGF-D is most abundant in the fetal lungs, while in adults, it is found in the muscle cells, lungs, heart and intestine. It acts through VEGFR-3 (similarly to VEGF-C), protecting the cells of the lymphatic endothelium; and by binding to the VEGFR-2 receptor, it also preserves the cells of the vascular endothelium [40].

Interestingly, EG-VEGF angiogenic effects on endothelial cells and vascular growth stimulation, proliferation and migration, along with increased vascular permeability are observed only in organs in which this protein is expressed. The presence of EG-VEGF in the fetal capillaries and the umbilical blood vessels shows its significant influence on the formation of placental vessels and facilitating the umbilical blood flow between the mother and the fetus [35,37].

Although PlGF does not promote vasodilation or proliferation, it does influence angiogenesis. The presence of this factor has been observed in trophoblast cells, in endometrium and in the cells of heart muscle, lungs and skin. It acts by indirectly displacing VEGF-A from VEGFR-1, allowing VEGF-A to bind to VEGFR-2 [35]. PlGF demonstrates a cytoprotective effect for microvascular retinal endothelial cells through VEGFR1/Akt signaling and can also promote uterine microvascular endothelial survival, along with VEGF-A and VEGF-C [40].

#### 2.1.2. Ligands Pathological Role

The family of vascular endothelial growth factors influences the process of blood vessel formation within a growing tumor. These proteins promote tumor growth and enable metastasis. The increase in their expression correlates with the progression of neoplastic disease. Many studies on various types and locations of neoplasms have shown the effect of high tissue expression of VEGF family factors and their receptors on the development of cancer, especially breast, cervical, colorectal and endometrial [37,42,43]. Elevated VEGF expression have been observed in other conditions associated with vascular proliferation, such as atherosclerosis, hemangiomas, liver and kidney diseases, chronic inflammatory diseases, skin and mucosa diseases, along with retinopathy. There are also states associated with inhibition of the angiogenesis process, in which a decrease in the expression of tissue VEGF factors was observed, e.g., ischemic disease, coronary artery disease, peripheral vascular disease, leukoencephalopathy and other brain diseases [37].

VEGF-A, due to increasing permeability of microvessels to circulating microparticles, is important in the course of pathological angiogenesis in malignant tumors, wounds and chronic inflammations, as well as in the course of ascites and pleural effusion. Its increased expression was observed in most cancers and was closely correlated with disease progression. VEGF-A by activating Src and disrupting the VE-cadhedrin/β-catenin complex weakens the endothelial barrier and facilitates the migration of tumor cells out of the vessel. Moreover, in the liver, it contributes to the breakage of hepatocellular tight junctions, which may lead to cancer invasion. VEGF is highly expressed by epidermal keratinocytes in wound healing and psoriasis. Local production of VEGF-A has been documented in arthritic synovial tissue and it likely correlates with disease progression in humans. VEGF-A has been shown to have an essential role in the pathogenesis of rheumatoid arthritis in animal models. Increased levels of VEGF-A were detected in tissues and samples from patients with asthma, and its high concentration correlate with the severity of the disease and poor condition of the respiratory tract. VEGF is postulated to contribute to asthmatic tissue swelling by increasing vascular permeability. VEGF-A also enhances respiratory sensitization and TH2-mediated inflammation (T-helper type 2) and influences the recruitment of dendritic cells. VEGF has fundamental role in several eye diseases, and its levels are elevated in the vitreous and retina of patients with active neovascularization for ischemic retinopathies—proliferative diabetic retinopathy, central retinal vein occlusion and preterm retinopathy. Moreover, VEGF165 promotes the survival of neurons during hypoxia by binding VEGFR-2 and NRP-1 [38,44].

VEGF-B expression is increased in breast, ovarian, colorectal, renal and prostate cancer, but only one study has addressed the role of VEGF-B in tumor development using a genetic model [41,45]. VEGF-B was found to significantly induce remodeling of the tumor microvasculature, which creates highly permissive conditions for tumor cell invasion and metastasis, independently from a VEGF-A [46].

It has been proved that VEGF-C and VEGF-D contribute to the formation of lymphatic metastases in papillary thyroid carcinoma by enhancing lymphangiogenesis [47]. Studies show that VEGF-C could be used as a predictive factor in diagnosed squamous cell carcinoma of the oral cavity and related metastases [18]. In malignant squamous cell carcinoma of the esophagus, it can be considered a reference indicator of metastases to the lymphatic system as its overexpression is significantly associated with infiltration of nearby lymph nodes [37,48]. In patients with gastric cancer, studies have shown similar possibilities for the use of VEGF and confirm that high concentrations of VEGF-C and VEGF-D indicate an unfavorable prognosis of survival [37,49].

### 2.2. Receptors

**Figure 4:**
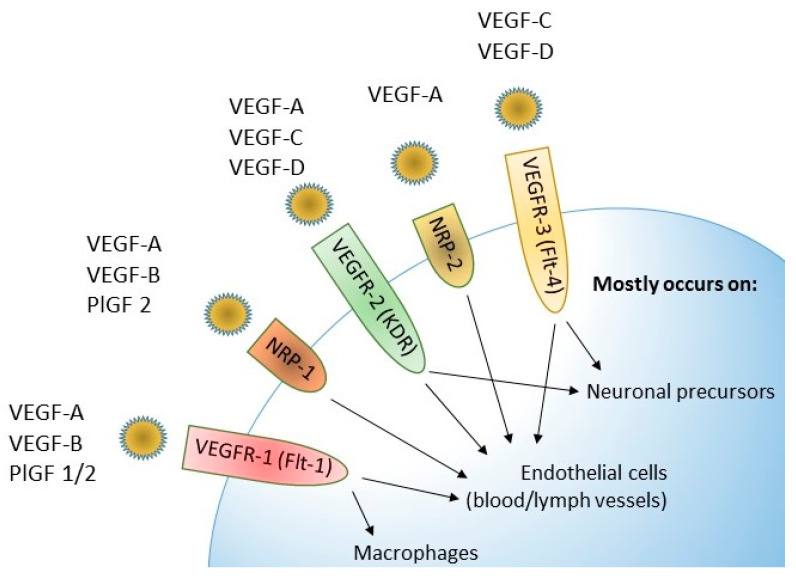
Possible bindings between VEGF family members ligands and receptors.

Three VEGF receptors are known: VEGFR-1, VEGFR-2 and VEGFR-3. Each of them consists of three domains—extracellular, transmembrane and intracellular. The binding of factors from the VEGF family to the extracellular domain leads to the activation of the enzyme called tyrosine kinase in the intracellular domain. Tyrosine kinase enables the phosphorylation of tyrosine residues, thanks to which signaling pathways inside the cell are activated, e.g., routes ERK and PI3K/Akt. Neuropilins-NRP-1 and NRP-2 are also mentioned in the course of angiogenesis [35]. The possibility of binding ligands to individual receptors for vascular endothelial growth factors is presented in Figure 4.

VEGFR-1, also known as Flt-1, belongs to the RTK family (receptor tyrosine kinases), and its molecular weight is 180 kDa. Some sources report that VEGFR-1 bound PlGF and enhances the release of VEGF-A, which activates VEGFR-2, and is leading to stimulation of cell migration and proliferation via PI3K/Akt, MAPK/ERK and other signaling pathways. Activation of this receptor also leads to the secretion of inflammatory cytokines such as TNF-α, MCP-1, IL-1β, 6.8 and MIP-1β, which additionally confirms its significant role in the process of pathological angiogenesis [35].

VEGFR-2, also referred as KDR, is the dominant receptor with a molecular weight of approximately 200–230 kDa. It has a stronger affinity for VEGF-A than for VEGF-C and VEGF-D. VEGFR-2 exhibits higher tyrosine kinase activity than VEGFR-1. Binding of VEGF-A to VEGFR-2 leads to activation of the PLCγ/PKC pathway as well as Ras/Raf/ERK/MAPK and PI3K/Akt, which affects both physiological and pathological angiogenesis [35,49].

Another receptor belonging to the same family is VEGFR-3, also known as Flt-4, with a molecular weight of 195 kDa. The signaling pathways responsible for activation of VEGFR-3 are PKC and Ras, as well as Akt/PKB. VEGF-C has the greatest influence on the activation of this receptor [35].

Neuropilins are endothelial transmembrane receptors with a molecular weight of 120–135 kDa that modulate RTK activity. They are able to bind to VEGF-A isoforms as co-receptors of which NP-1 has a highest affinity to VEGF165, and NP-2 has a highest affinity to VEGF-C, VEGF-D and VEGFR-3 [35,37,49].

#### 2.2.1. Receptors Physiological Role

VEGFR-1 is found mainly on the surface of vascular endothelial cells, but also to a lesser extent on monocytes/macrophages, marrow progenitor cells, cancer cells, mesangial cells, vascular smooth muscle and trophoblastic muscle cells. It has been proven that VEGFR-1 is not active during embryonic vasculogenesis due to the lack of proproliferative activity [35].

VEGFR-2 is present mostly on endothelial cells of blood and lymph vessels, also on the surface of megakaryocytes, osteoblasts, neuronal, cancerous, hematopoietic cells, pancreatic duct and retinal progenitor cells. Due to the significant influence on cell proliferation, increased KDR expression plays an important role in the course of vasculogenesis. VEGFR-2 has an anti-apoptotic effect, and also activates integrins, which intensify cell migration. Activation of the Akt protein kinase translates into vasodilation by the formation of endothelial nitric oxide synthase eNOS, which enables the formation of vasodilating nitric oxide. Moreover, activated VEGFR-2 stimulates the secretion of von Willebrand factor (vWF) by the endothelium [35,49].

VEGFR-3 has high affinity to VEGF-C and VEGF-D, and effect on lymphangiogenesis in both embryonic development and pathological conditions. Its expression is observed on endothelial cells of lymphatic vessels, but also on osteoblasts, macrophages and neuronal precursors. Activated VEGFR-3 contributes to the proliferation, migration, differentiation and survival of lymphatic endothelial cells. It is also believed that it plays an important role in the initiation of primary lymphedema and is involved in the formation of distant metastases by the lymphatic route [35].

Neuropilin 1 is found mainly on the endothelial cells of the arteries, but also on the surface of neurons, cancer cells and smooth muscle cells. It affects the activity of VEGFR-2, thus enhancing the processes of migration and angiogenesis. Neuropilin 2 is expressed on lymphatic endothelial cells and through binding to VEGF-C it enhances the action of VEGFR-3 [35,37,49].

#### 2.2.2. Receptors Pathological Role

VEGFR-1 has a 10-fold higher affinity for VEGF-A than VEGFR-2, which results in its strong influence on the migration of endothelial and inflammatory cells in the course of pathological angiogenesis [35]. VEGFR-1 expression is raised in several tumor types and lead to invasion and resistance to anti-VEGF-A therapies. The levels of sVEGFR-1 appear to play a significant role in cancer progression—the VEGF-A/sVEGFR-1 ratio detected in tumor tissue, serum or plasma correlate with metastasis, malignancy grade, survival and therapy response [50,51,52,53]. VEGFR-1 higher levels were observed in breast cancer, but also in renal and lung cancer, high-grade gliomas, mesothelioma and highly aggressive osteosarcoma. Moreover, VEGFR-1 and -2 could potentially become prognostic markers for hepatocellular and lung carcinoma. VEGFR-1 expression is also observed in pancreatic carcinoma [52,54]. Plasma sVEGFR1 and sVEGFR2 levels are significantly decreased in patients with Alzheimer disease [55] and their elevated expression correlates with worse outcome in breast cancer patients [54]. It was suggested in the research of Gumus et al. [56] that VEGFR-2 significantly influences the pathogenesis of pterygium, and its overexpression in this disease indicates a higher probability of postoperative recurrence. In case of VEGFR-3 and VEGF-C, a significant number of studies were conducted. The dominant part of these studies concern breast cancer [57,58,59,60,61], but gastric, lung and ovarian cancer are addressed as well [62,63,64,65].

## 3. Role of VEGF in Angiogenesis

Angiogenesis is a complex process that concerns the formation of new capillaries. Its regulation is associated at the molecular level with receptors, growth factors, humoral factors and extracellular matrix (ECM) proteins. The process of creating new vessels can occur both in physiological conditions related to tissue regeneration, embryo development and wound healing, as well as in pathological conditions such as cancer, ischemia, inflammation and atherosclerosis. Angiogenesis is crucial in the progression of a malignant tumor due to the supply of nutrients and oxygen to tumor tissues, as well as the spread of cancer cells via blood vessels [43]. An important factor influencing the angiogenesis process is the state of hypoxia, which is caused by factors such as hypoglycemia, hypertension and chronic inflammation. During hypoxia, endothelial cells (EC) and smooth muscle cells (SMC) secrete proteins with proangiogenic properties such as VEGF [35,66]. These cells are equipped with a variety of mechanisms that detect changes in oxygen pressure—NADPH oxidase, nitric oxide synthase (eNOS) and hemoxygenase. In all tissues, including vascular structures, the response to changes in oxygen pressure is also exerted by the hypoxia-inducible factor (HIF). All HIF isoforms are capable of heterodimerization with the ligand-activated aromatic hydrocarbon receptor dimer with nuclear translocating protein (ARNT), resulting in an active transcription complex that initiates the expression of genes that are responsible for the regulation of metabolism and angiogenesis [66]. In the classical model of angiogenesis, initially, the permeability of the vascular walls is increased and the pericytes lying along them are partially detached by proteinases. At the same time, hypoxia determines the increase in the expression of the HIF-1 protein, which results in the secretion of pro-angiogenic factors, e.g., VEGF, FGF-2, PDGF-β, TGF-β Ang-1, Ang-2 and TNF-α. Those factors can bind to specific receptors on the surface of the endothelium, which re-activates many signaling pathways. Activated matrix metalloproteinases (MMPs), mainly MMP-9, MMP-3 and MMP-2, cause degradation of the extracellular matrix and basement membrane, which allows endothelial cells to migrate to the perivascular area towards the VEGF gradient and proliferate. The αvβ3 integrin expressed in endothelial cells facilitates the adhesion of endothelial cells to the extracellular matrix and their migration [36]. Endothelial cells differentiate into various types, including stalk cells, tube cells and tip cells (also known as frontal cells or sprouting cells) capable of recognizing the concentration gradient of pro-angiogenic factors. Frontal cells expressing DLL4 do not proliferate; they are only responsible for the direction of new vessel formation. It is thanks to stalk cells expressing Notch-1, which have proliferating properties, that a new vascular structure is formed. Tube cells do not have the ability to proliferate, their function is to shape the final appearance of the vessels. After the initiation of blood flow through the new vessels, the basement membrane is synthesized, proliferation and migration are inhibited, and endothelial cells release PDGF-β, which has a chemotactic effect on pericyte precursors, binding to endothelial cells and differentiating into pericytes [35,36,43]. The course of angiogenesis is also affected by its inhibitors, which prevent the formation of vessels or affect their removal by affecting pro-angiogenic particles, including VEGF, basic fibroblast growth factor (FGF2), transforming growth factor (TGF-α and TGF-β), tumor necrosis factor (TNF-α), placental growth factor, platelet endothelial growth factor, angiogenin, interleukin 8 (IL-8), epidermal growth factor, granulocyte colony stimulating factor, and hepatocyte growth factor. It is extremely important to maintain vascular homeostasis, which is determined by the balance between the secretion of activators and inhibitors of angiogenesis [66]. In the course of neoplastic changes, cells may acquire an angiogenic phenotype. A group of tumor cells with this phenotype have specific mutations and are capable of producing neovascular initiation factors and/or inhibiting angiogenesis inhibitors. In addition, cancers are closely associated with inflammation. This process increases vascular permeability, which causes an increase in interstitial pressure in the tumor tissue and is one of the conditions for the initiation of VEGF-induced neovascularization. Moreover, the resulting leak in the vessels allows cancer cells to expand along with the bloodstream to distant sites and form metastases [36].

## 4. Possible Utility of the VEGF Family Members in Breast Cancer

### 4.1. Diagnostic Biomarkers

Tumor stromal VEGF-A expression is a valuable prognostic indicator during diagnosis and can therefore be used to divide inflammatory breast cancer patients into low- and high-risk groups for death and relapses. Determination of tumor stromal VEGF-A may be useful for identifying patients in whom anti-angiogenic treatment would be beneficial [67]. In the study of Zajkowska et al. [45], diagnostic utility of VEGF-A concentration was higher than CA 15-3, especially in the early stages of breast cancer; therefore, it is believed that the combined analysis of both parameters could be used for the early detection of this disease. High expression of PlGF and VEGF-A in tumor tissue was associated with significantly shorter recurrence-free survival (RFS), which is why further investigation as prognostic markers in breast cancer could be beneficial [68]. In the research of Zajkowska et al. [45], VEGF-B achieved the highest sensitivity and NPV in the group of breast cancer patients among other studied proteins. However, more studies are needed to compare the results and determine its diagnostic usefulness.

The study of Ying-Chun Zhao et al. [69] revealed that in breast cancer, a large number of lymphatic vessels are present and the presence of metastases to the lymph nodes and other organs is the result of increased VEGF-C expression. With the inhibition of VEGF-C and VEGF-D or VEGFR-3 in experimental models, the process of new lymphatic vessels formation and growth of existing capillaries is inhibited, and the number of metastases and lymph nodes involved is reduced, which positively affects the patient’s survival. Sentinel node lymphangiogenesis has been shown to occur earlier than metastasis to other organs due to VEGF-C secreted by primary breast tumor cells and then transported by lymphatic vessels to the sentinel node [69]. In in vitro studies, VEGF-C and MT1-MMP, which increases the expression of VEGF-C in human breast cancer, increased the invasiveness of cancer cells and lymph node metastasis [70]. Additionally, VEGF-C has the potential to be a better tumor marker than CA 15-3 (currently considered the most important breast cancer marker), especially in early TNM stages-I and II [61]. In one of the meta-analyses, it was found that VEGF-C overexpression significantly affects the reduced survival of breast cancer patients and determines its concentration may be an important step in treatment optimization [71].

Some studies showed that VEGF-D serum concentration (followed by high sensitivity) and tissue expression in BC patients were increased; however, there are not enough studies to clearly state its utility in diagnostic fields [59,61].

PlGF protein supplementation in vitro promoted migration, invasion and adhesion of metastatic breast cancer cells to bones [72], as well as induced proliferation of breast cancer [73]. High PlGF levels correlate with poor prognosis [74] and recurrence, metastasis and patient mortality [75], as well as shorter recurrence-free survival [73].

### 4.2. Therapeutic Targets

Over the past decades, VEGF and their receptors have been identified as a strategic target of modern cancer therapies mostly by inhibition of VEGF/VEGFR signaling pathway. Inhibition has been achieved through blocking ligand binding to the extracellular domain of the kinase receptor with monoclonal antibodies and preventing the activation of VEGFR-2 receptors using tyrosine kinase inhibitors (RTKIs) [76].

Bevacizumab is a humanized monoclonal antibody blocking the interaction of VEGF ligands to VEGFR-2 approved in 2004 for the treatment of metastatic colorectal cancer and then for breast and lung cancers as well. Treatment of metastatic breast cancer with Paclitaxel combined with Bevacizumab increases progression-free survival but does not extend overall survival. The most effective clinical use of angiogenesis inhibitors is likely to be in patients with micrometastatic disease as adjuvant therapy [77]. However, addition of Bevacizumab to neoadjuvant therapy increased a number of toxic effects—increased hypertension, left ventricular systolic dysfunction, the hand–foot syndrome, and mucositis. The US FDA had revoked the indication of Bevacizumab to treat patients with metastatic breast cancer in 2011 due to negative outcomes, which outweigh positive effects [78,79,80,81,82]. Ramucirumab is a monoclonal antibody targeting VEGFR-2 [83] firstly used as monotherapy for the treatment of metastatic gastric cancer [84], then also in combination treatment with chemotherapy for gastric cancer, non-small cell lung cancer (NSCLC) and colon cancer [85]. Some studies investigated relevance of Ramucirumab in breast cancer treatment, however, without acceptable results [86,87]. Sorafenib is multikinase inhibitor of VEGFR, PDGF receptor (PDGFR), and Raf used in the treatment of renal cell carcinoma, HCC and thyroid cancer [88]. Sorafenib in breast cancer showed modest potential in early clinical trials, but later studies repeatedly showed lack of survival advantage for the combination of Sorafenib and chemotherapy in patients with advanced breast cancer [89,90]. Sunitinib is multitargeted thyrosine kinase inhibitor of VEGFR-1, VEGFR-2, FLT-3, c-KIT, PDGFR-α and PDGFR-β. Sunitinib is approved for the treatment of advanced renal cell carcinoma, gastrointestinal stromal tumors [91] and pancreatic neuroendocrine tumors [92]. Clinical studies for the efficacy of Sunitinib in breast cancer treatment were not satisfying so far [93,94,95]. The studies on Vandetanib, Axitinib, Pazopanib and Cediranib were also not relevant for a better treatment of breast cancer [96,97,98].

Novel approaches for the treatment of aggressive and advanced breast tumors include targeting pericytes, use of miRNAs and extracellular tumor-associated vesicles, immunotherapeutic drugs and nanotechnology [79]. Targeting pericytes enables the normalization of tumor vasculature and prevents cancer cell metastasis, improves anticancer therapies and improves recognition by the patient’s immune system. In some types of cancer, high pericyte coverage of the tumor vessels impairs anti-angiogenic therapies. However, low pericyte coverage leads to the reduction of vascular stability and increases permeability, which allows cancer cells to metastasize. The targeting pericytes strategy may be crucial to normalizing tumor vascularity; however, the negative effects of lack of pericyte coverage should be evaluated [99,100].

Innovatory methods using microRNAs may suggest a novel therapeutic approach in breast cancer treatment, although further studies including animal models are needed [101]. MicroRNAs are critical regulators of angiogenesis signaling pathways involved in tumor metastasis by interacting with target mRNAs [102]. The present well-described groups of miRNAs are involved in the regulation of endothelial cell function and angiogenesis, making them an attractive target for anti-cancer therapies. Interestingly, miRNAs can be transported between cancer cells and stromal cells through extracellular vesicles known to mediate cell-to-cell communication in the tumor microenvironment [103]. In the research carried out so far miRNA-153 suppressed breast tumor angiogenesis through targeting HIF-1α and Ang-1 [104], miRNA-140-5p inhibited tumor invasion and angiogenesis by silencing VEGF-A [105] and miRNA-29b inhibited proliferation, migration and tube formation of endothelial cells [106].

Normal vasculature is essential for immunosurveillance and termination of cancer cells by immune system. Disorganized tumor vessels create a barrier blocking functionality of immune cells, especially the cytotoxic T lymphocytes. Selective targeting of adhesion molecules and normalization of tumor vasculature could improve immune cell endothelial adhesion and enhance positive immune response in breast cancer [107]. In a preclinical study performed by Allen et al. [108], it was found that the use of a combination of anti-VEGFR-2 and anti-PD-L1 antibodies in the treatment of breast cancer made tumors more sensitive to the therapy and prolonged its effectiveness, while the study Li et al. [109] showed that the combination of low doses of anti-VEGFR2 antibody with protein-1 (PD-1) anti-programmed cell death therapy normalized tumor vascularization, induced cell infiltration immune system and increased PD-1 expression on cells of the immune system in a mouse model of breast cancer. Studies performed with use of the combination of Apatinib and anti-PD-1 in therapy have shown promising results [105]. Numerous clinical trials combining anti-angiogenic elements with inhibitors of the immune checkpoints are proceeding [99].

Nanotechnology is considered to be the future of tumor vascularization therapies that could significantly improve the action of anti-angiogenic drugs and facilitate their accumulation in neoplastic tissue [110]. In vitro and in vivo tests showed that quercetin conjugated to gold nanoparticles inhibited angiogenesis and breast cancer invasion by targeting the EGFR/VEGFR-2 signaling pathway [111]. Moreover, the radical-containing nanoparticles increased the anti-angiogenic activity in breast cancer [112]. In the study of Gong et al. [113], nanoparticles delivering a sphingosine-1 phosphate receptor inhibitor drastically inhibited triple negative breast cancer growth and angiogenesis in vivo. Summary of the described data is included in Table 2.

The usefulness of proteins from the VEGF family in the course of breast cancer has not been fully explained. Currently, in the available literature, works that describe the usefulness of these parameters as markers and therapeutic targets in the course of breast cancer can be found. Current reports suggest that further research on discovering and expanding knowledge of the VEGF family may be extremely useful. Although it has been noted that some of the examined parameters have extremely high diagnostic sensitivity, especially in the early stages (TNM stage I and II) of the disease, studies should be continued on their diagnostic usefulness in combination with more organ-specific parameters. In the case of the usefulness of VEGF as therapeutic targets, more papers describing the current state of knowledge are available. As described, anti-VEGF therapy using monoclonal antibodies is not a first choice therapy due to possible side effects. However, there is much hope that the process of angiogenesis and the VEGF factors involved in it are still considered an important element in the development of BC and are taken into account when conceptualizing new therapeutic methods, e.g., miRNA therapy or nanotechnology by silencing of VEGF or targeting VEGF pathways.

## 5. Conclusions

The VEGF family includes the most important growth factors from both pathological and physiological angiogenesis point of view. VEGF proteins have the potential to become early biomarkers of breast cancer, indicators of treatment success and patients survival. Moreover, inhibition of angiogenic signaling pathways may be a future direction of anti-cancer therapies. Chemotherapy combined with anti-angiogenic elements, nanotechnology, immunotherapeutic drugs or miRNAs, and targeting pericytes may increase the survival rate of patients with diagnosed breast cancer. This indicates the possible contribution and usefulness of VEGF family ligands and their receptors in this malignancy.

## Figures and Tables

**Table 1:** The frequency of follow-up in asymptomatic women with no risk factors for breast cancer.

Women’s Age	Palpation Examination Performed by Specialist	Mammography
20–39	Every 36 months	No
40–49	Every 12 months	No
50–69	Every 12 months	Every 24 months
>70	Every 12 months	No

**Table 2:** The summary of VEGF ligands and receptors usefulness in detection and therapy of BC.

Parameter	↑ Expression	↑ Concentration	Therapy
VEGF-A	qualification to anti-angiogenic therapy associated with ↓RFS	early detection of BC higher SE than CA 15-3 in early BC stages	binding to VEGFR-2 blockade silencing with miRNA-140-5p
VEGF-B	no data available	higher SE and NPV than CA 15-3	no data available
VEGF-C	associated with lymph node and distant metastases ↑ invasiveness ↓ survival	early detection of BC higher SE than CA 15-3 in early BC stages	inhibition connected with ↓ number of involved lymph nodes and metastases ↑ survival
VEGF-D	associated with lymph node and distant metastases	high SE in BC	
VEGFR-1	higher expression in BC	altered in BC considerable SE with CA 15-3	blockade of VEGF ligands binding (e.g., sunitinib)
VEGFR-2	higher expression in BC	not suitable for detection of BC	blockade of VEGF ligands binding (e.g., bevacizumab, ramucirumab) nanotechnology (quercetin)
VEGFR-3	associated with lymph node and distant metastases	suitable for detection of BC higher SE than CA 15-3 in all BC stages	inhibition connected with ↓ number of involved lymph nodes and metastases ↑ survival
PlGF	associated with ↓ RFS	↓ prognosis ↑ recurrence ↑ metastasis ↑ mortality	no data available

Abbreviations: ↑—raised; ↓—lowered; RFS—reccurence-free survival; BC—breast cancer; SE—diagnostic sensitivity; NPV—negative predictive value.

## Data Availability

Not applicable.
